# Dysentery

**Published:** 1890-07

**Authors:** A. Garwood

**Affiliations:** New Braunfels


### DYSENTERY.

BY A. GARWOOD, M. D., NEW BRAUNFELS.

Read at Austin District Medical Society, June 19, and ordered published in the official organ.

MY SUBJECT, acute dysentery, is as old as any I might have selected, and yet, as to cause and treatment, is as fruitful of discussion.

I deem it a practical and proper subject, because within the next few weeks it will become an every day disease, and, you will agree, that advance discussion of life-giving methods is better far than an after comparison of mortality lists.

Taking it for granted that each one present is a more diligent student than myself, I will not attempt to shed "the electric light of science" upon the interesting question of etiology.

I trust, however, that those present who have more positive knowledge than myself as to the special cause, will enlighten us with their views. Books tell us that "dysentery is classed among the filth diseases, and filth is supposed to produce its effects chiefly by favoring the production and multiplication of specific poisons of a living, or at least, organic character." Organic, and particularly animal matter, in a state of decomposition with certain climatic influences, is among the causes of disease. An attack may be precipitated by any one of general co-operating causes, as eating unripe fruit, great fatigue, catching cold, malaria, etc.

The disease, *per se*, is an inflammatory lesion of the large intestine, characterized by mucus and bloody stools, accompanied usually by tenesmus. The two forms, sporadic and epidemic, are essentially the same, though the bowel lesion is, as a rule, greater and more disorganizing in effect in the epidemic variety. The large intestine may be the seat of a catarrhal or croupous inflammation. The mucous membrane may be swollen, and the secretion of the mucus greatly increased and mixed with blood. The croupous form of the disease is usually more serious than the catarrhal, the local symptoms being more pronounced, the passages more frequent, with a greater amount of blood.

The pain and straining are more severe, and fever is well marked, with full and rapid pulse. There is also a follicular form of the disease, wherein the follicles become swollen, and ultimately small round ulcers are formed. This form hardly concerns us, and in treatment will not be considered.

Symptoms and diagnosis are too familiar to all, so I pass to a consideration of the treatment, which, I may as well state in the beginning, has not yet crystalized into any definite shape.

The first step in treatment is to see that the bowels are relieved of all irritating fecal contents. Usually a preceding diarrhœa has accomplished this for us, but it is not always safe to presume so; for, even though we are called to the case several days after its inception, we may find hardened scybala in the colon, and keeping up the irritation we are called upon to relieve. Obviously, at such time an opiate would do more harm than good, yet I fear that in our anxiety to relieve pain and retain the confidence of our patient we are too prone to a too early use of this drug.

A good dose of castor oil or sulphate of magnesia, or a mild enema, is indicated.

I usually employ, at once, a large injection of a 1 to 2% solution of creolin. By this method all foul and irritating matters are removed from the intestine, and the supposed micro organism may me rendered less active.

It may not be out of place to here remark that, in giving the enema, a soft tube should be used, and the patient, if possible, should assume the knee-chest position. In children or enfeebled subjects, tte buttocks should be elevated by means of a firm pillow. The quantity of injection should vary from one to six pints, to be repeated two or three times a day, according to the number and character of the discharges. I give creolin enemata the preference, because it is a safe disinfectant, and is less toxic than either carbolic acid or bichloride of mercury. It is also free from irritating local effects, and has a desirable hæmostatic action on the intestinal wall. Added to this, it is cheap, and far pleasanter in every way to handle than the acid or bichloride. If, after the enema, there remains much tenesmus or abdominal pain, we may give an opiate, or, in children, use ice water injections, or give 10 to 15 drops of a 10% solution of cocaine added to a teaspoonful of water per rectum. Repeat two to three times a day, as indicated.

By strictly confining the patient to bed and treating the disease locally, I believe most mild cases may be cured without a dose of medicine per orem.

The diet, of course, must be restricted to the blandest and most digestible articles, while, if the vital powers depreciate, alcoholics must be freely administered.

The ipecac treatment has many advocates, and is said to have reduced mortality in India from 80 to 20 per one thousand. I have experienced, in a few cases, most satisfactory results from its use. In fact, I believe it seldom fails to benefit the patient so far as the flux is concerned, but in my experience, nausea and distressing vomiting so often occur that the remedy, with me, is not a favorite one.

I have failed oftener than succeeded to avoid its emetic effects, and this after the most approved method of administration. I have thought that the fluid extract per rectum might serve us better, but have never given it a trial. I usually employ beta napthal as an intestinal disinfectant, and, according to indications, use bismuth submit. (lg. doses), belladona, aconite, opium, calomel, turpentine, quinine, etc.

Coupled with the medicinal treatment, thorough cleanliness of the patient, his bed, linen, etc., must be insisted upon. Disinfection and immediate removal of the stools and free ventilation of the sick room, should be enjoined.

